# Three-dimensional human bile duct formation from chemically induced human liver progenitor cells

**DOI:** 10.3389/fbioe.2023.1249769

**Published:** 2023-08-21

**Authors:** Peilin Li, Daisuke Miyamoto, Yu Huang, Tomohiko Adachi, Masaaki Hidaka, Takanobu Hara, Akihiko Soyama, Hajime Matsushima, Hajime Imamura, Kengo Kanetaka, Weili Gu, Susumu Eguchi

**Affiliations:** ^1^ Department of Surgery, Nagasaki University Graduate School of Biomedical Sciences, Nagasaki, Japan; ^2^ Department of Surgery, Guangzhou First People’s Hospital, School of Medicine, South China University of Technology, Guangzhou, Guangdong, China

**Keywords:** chemically induced progenitor cell, cholangiocyte, bile duct regeneration, bile canaliculi, hepatic organoid

## Abstract

**Background:** The intrahepatic bile ducts (BDs) play an important role in the modification and transport of bile, and the integration between the BD and hepatocytes is the basis of the liver function. However, the lack of a source of cholangiocytes limits *in vitro* research. The aim of the present study was to establish three-dimensional BDs combined with human mature hepatocytes (hMHs) *in vitro* using chemically induced human liver progenitor cells (hCLiPs) derived from hMHs.

**Methods:** In this study, we formed functional BDs from hCLiPs using hepatocyte growth factor and extracellular matrix. BDs expressed the typical biliary markers CK-7, GGT1, CFTR and EpCAM and were able to transport the bile-like substance rhodamine 123 into the lumen. The established three-dimensional BDs were cocultured with hMHs. These cells were able to bind to the BDs, and the bile acid analog CLF was transported from the culture medium through the hMHs and accumulated in the lumen of the BDs. The BDs generated from the hCLiPs showed a BD function and a physiological system (e.g., the transport of bile within the liver) when they were connected to the hMHs.

**Conclusion:** We present a novel *in vitro* three-dimensional BD combined with hMHs for study, drug screening and the therapeutic modulation of the cholangiocyte function.

## 1 Introduction

The liver consists of two types of endodermal epithelial cells, hepatocytes, and biliary epithelial cells (BECs), termed cholangiocytes, which differentiate from hepatoblasts during development (Tanimizu et al., 2013; O'Hara et al., 2013). The BECs form bile ducts (BDs) that connect the liver and the intestine to secrete bile, which is generated in hepatocytes, into the intestine (Alpini et al., 2002). BECs modify and transport the bile produced by hepatocytes so that they can protect the liver from bile-induced damage (O'Hara et al., 2013). The homeostasis of the connection between the BDs and the hepatocytes is therefore crucial for maintaining a normal liver function and preventing liver damage or disease (Cao et al., 2017). Functional impairment of BECs and the transportation of bile acids play an essential role in the development of various types of biliary disorders and liver failure, which can eventually only be treated by liver transplantation (Strazzabosco et al., 2005). However, the physiology and pathophysiology of cholangiopathies have not yet been fully elucidated (Strazzabosco et al., 2005; Lazaridis and LaRusso, 2015). This is mainly due to the lack of relevant *in vivo* and *in vitro* models for the study of biliary tract development, cholangiopathies and drug assays, especially for bile transport and drainage between BECs and hepatocytes in humans. The shortage of the cell source also limits the *in vitro* study of human BECs (hBECs) and the three-dimensional structural function of the BD (Buisson et al., 2019).

It has been proven to be feasible to use isolated BECs or stem cells as a source to establish a three-dimensional biliary network *in vitro* by bioengineering methods (Ramli et al., 2020; Huang et al., 2021; Sato et al., 2021; Roos et al., 2022; Wang et al., 2022; Yan et al., 2022). Currently, there has been significant improvement in the generation of functional hepatocytes from induced pluripotent stem cells (iPSCs). Several *in vitro* models of human hepatic disease have been established based on iPSCs (Olgasi et al., 2020; Kim et al., 2022; Park et al., 2022; Xu et al., 2022). Some researchers have also used chemically induced pluripotent stem cells to build various hepatocyte and cholangiocyte organoids (Si-Tayeb et al., 2010; Chen et al., 2018; Aizarani et al., 2019; Ramli et al., 2020; Wang et al., 2020; Carberry et al., 2022). Although there has been significant success in the induction of BECs and BD cysts from animal cell sources, the induction of human three-dimensional BDs (hBDs) has not fully progressed (Huang et al., 2020; Ramli et al., 2020; Huang et al., 2021). iPSCs or liver progenitor cells (LPCs) constitute BD epithelial cells, spheroids and biliary tubules in different cultural environments needed the different combinations of growth factors (Tanimizu et al., 2007; Tian et al., 2016). However, iPSCs with altered gene sequences and the isolation of BECs with limited sources still have limited clinical applications, although BECs and biliary organoids have shown great value in the treatment of biliary disease (Sampaziotis et al., 2021; Velazquez and Ebrahimkhani, 2021). Additionally, the present studies of BDs constructed *in vitro* usually ignore the relationship between BDs and hepatocytes. Hepatotoxicity studies based on hepatocytes are basically studies of the hepatocyte culture models or cystic hepatic organoids (Li et al., 2022; Park et al., 2022). Cystic organoids contain BECs and hepatocytes and exhibit corresponding characteristic cell functions. The multicellular tissue of BD combined with hepatocytes is a hepatic organoid that can represent the physiological state.

Katsuda et al. used a small molecule cocktail to chemically induce rodent mature hepatocyte (rMH) dedifferentiation into chemically-induced liver progenitor cells (CLiPs) with bidirectional differentiation potential of MHs and BECs, and on this basis, they and other researchers developed methods for human MHs (hMHs) (Katsuda et al., 2017; Katsuda et al., 2019; Kim et al., 2019). Human CLiPs (hCLiPs), chemically induced from hMHs, offer an appreciated cell source for regenerative medicine (Katsuda et al., 2019). The dilative and proliferative ability and bidirectional differentiation potential of hCLiPs bring considerable prospects for the treatment of end-stage liver disease. Huang et al. established biliary duct-like structure integrated hepatocyte tissues from rCLiPs, providing an excellent *in vitro* model for hepatobiliary disease research but not for humans (Huang et al., 2020; Huang et al., 2021). The BD-hepatocyte connected tissue as a cocultured organoid model provides a model for studying the integrated BD-hepatocyte and hepatocyte-cholangiopathy *in vitro*, which can be used to study the transport of bile and establish *in vitro* disease models.

In this study, the primary aim was to utilize hCLiPs to establish functional hepatic tissue with a three-dimensional BD connected to the hMHs. The connected tissue exhibits both structural and functional characteristics similar to BD-hepatocyte transporters and bile canaliculi. The interconnected tissue was capable of performing the crucial functions of bile transportation, collection, and delivery.

## 2 Methods

### 2.1 Cell culture and conversion

Human Cryo-Hepatocytes (CHHs) (Lot.416, Corning, Woburn, MA, United States) were seeded into collagen type-I-coated dishes (Asahi Techno Glass, Tokyo, Japan) at a density of 2 × 10^4^ cells/cm^2^ in STIM medium to promote attachment to the plate surface. The STIM medium was a hepatocyte culture media kit with 10 ng/μL epidermal growth factor (EGF) containing 1x penicillin‒streptomycin-glutamine (100X) (Gibco™, Tokyo, Japan) and 10% fetal bovine serum (FBS, Gibco™, Tokyo, Japan). Four hours later, the culture medium was changed to small chemically reprogrammed culture medium. The small chemical reprogramming culture medium was DMEM/F12 containing 2.4 g/L NaHCO_3_ and L-glutamine (Life Technologies, Tokyo, Japan) and supplemented with 5 mM HEPES, 30 mg/L L-proline, 0.05% BSA, 10 ng/mL EGF (all from Sigma‒Aldrich Japan, Tokyo, Japan), insulin-transferrin-serine (ITS)-X (Life Technologies, Tokyo, Japan), 10−7 M dexamethasone (Dex) (Fuji Pharma Co. Ltd., Tokyo, Japan), 10 mM nicotinamide (Sigma‒Aldrich, Tokyo, Japan), 1 mM ascorbic acid-2 phosphate (Wako Pure Chemical, Osaka, Japan), 100 U/mL penicillin, and 100 mg/mL streptomycin (Life Technologies, Tokyo, Japan) in addition to two small chemical molecules of 0.5 μM A-83–01 (Wako Pure Chemical, Osaka, Japan), 3 μM CHIR99021 (A10199, AdooQ BioScience, Irvine, CA, United States) and 10% FBS, which would be called FAC medium. The culture medium was changed 1 day after seeding and every two/3 days thereafter. It takes 14–16 days to generate hCLiPs at up to 90% confluence from CHHs.

### 2.2 Removal of fibroblasts and subculture of hCLiPs

The cultured cells reached ≥90% confluence and were treated with TrypLE Express (Life Technologies, Tokyo, Japan) for 15–20 min. The hCLiPs were expanded with an equivalent volume of preculture medium, and the cells were transferred to a 15 mL conical tube and centrifuged at 220 *g* for 5 min. The cell pellet was resuspended in 5 mL of culture medium, and the total number of cells and percent viability were determined using a hemocytometer. The isolated hCLiPs were seeded onto gelatin-coated culture plates (Asahi Techno Glass, Tokyo, Japan) at a density of 5.0 × 10^4^ cells/cm^2^ and incubated for 60 min. The cells in the medium were isolated and seeded onto collagen-coated plates at a density of 5.0 × 10^4^ cells/cm^2^ and incubated for 10 min. After 10 min, the cells were isolated and seeded onto collagen-coated plates at a density of 1.0 × 10^4^ cells/cm^2^. The cells seeded onto the third collagen-coated plates achieved an appreciable purity. This method was drived from the published patent [JP2020-162551 (P2020-162551A)].

### 2.3 Human BD formation from hCLiPs

BDs were differentiated and formed from hCLiPs as previously reported (Huang et al., 2021). Briefly, 1–2 days before collecting the hCLiP suspension, we used embryonic fibroblast feeder cells (MEFs) (Cat #PMEF-N, Merck Millipore, Billerica, MA United States) to form an MEF feeder layer by seeding 1–2 × 10^5^ cells on collagen-coated 12-well plates (approximately 3 × 10^4^ cells/cm^2^) in DMEM containing 10% FBS. We plated the dissociated hCLiP suspension onto the MEF feeder layer at a density of 4–5 × 10^5^ cells/well (1.2 × 10^5^ cells/cm^2^) in FAC medium for cell attachment for 1 day. Thereafter, we replaced the medium with BEC induction medium (BIM), which was mTeSR™1 Complete Kit (Catalog #85850, STEMCELL Technologies, Tokyo, Japan), including mTeSR™1 basal medium supplemented with TeSR™1 5X supplement, with the addition three small chemical molecules of 10 µM Y-27632, 0.5 µM A-83–01, 3 µM CHIR99021, hepatocyte growth factor (HGF, Sigma‒Aldrich Japan, Tokyo, Japan) and EGF, every 2 days for 6 days, followed by BIM supplemented with 2% growth factor reduced Matrigel (Catlog 354230, Corning, Bedford, United States) for an additional 6–10 days, to facilitate the maturation of BECs and the formation of biliary structures.

### 2.4 Integrated BD structure to human hepatocytes

We plated CHHs onto three-dimensional BD at a density of 1 × 10^5^ cells/12-well plate (2.5 × 104 cells/cm^2^) for 1 day in hepatocyte-defined medium (Catlog 05449, Corning, Bedford, United States) supplemented with 10 μg/mL EGF and 10% FBS. We then replaced it with BIM supplemented with 2% Matrigel for another 2–4 days. The BD was automatically attached with hepatocytes with bile canaliculi to the biliary cells.

### 2.5 Gene expression analysis by quantitative reverse-transcription polymerase chain reaction (qRT‒PCR)

Samples were cultured in dishes under various conditions, and mRNA was extracted using a spin column (NucleoSpin RNA II; Macherey-Nagel, Düren, Germany). Synthesis of cDNA was performed using a high-capacity cDNA reverse transcription kit (Applied Biosystems, Tokyo, Japan). Samples were then stored at −20°C until their analysis by polymerase chain reaction (PCR), which was performed using an Applied Biosystems StepOne Plus Real-time PCR System with TaqMan Gene Expression Assay Kits (Applied Biosystems, Tokyo, Japan) according to the manufacturer’s instructions. Briefly, PCR mixtures contained 1 μL of cDNA, 1 μL of TaqMan Gene Expression Assay probe, 5 μL of TaqMan Fast Advanced Master Mix (both from Applied Biosystems), and 13 μL of nuclease-free water. All TaqMan gene primers are listed in Supplementary Table S1. The thermocycling conditions were 95°C for 20 s followed by 40 cycles of 95°C for 1 s and 60°C for 20 s. Expression levels were quantified using the comparative cycle time method. Cycle threshold (Ct) values were automatically determined by the Applied Biosystems StepOne Plus Real-Time PCR System, and fold changes in gene expression were calculated by the 2^(−ΔΔCT) method. Expression levels were normalized to those of the housekeeping gene and internal control glyceraldehyde 3-phosphate dehydrogenase (GAPDH).

### 2.6 Immunofluorescence

Cultured cells were fixed with 4% paraformaldehyde in phosphate-buffered saline (PBS) (Wako Pure Chemical, Osaka, Japan) for 10 min. Fixed samples were then incubated in 0.1% Triton X-100 (Sigma‒Aldrich, Tokyo, Japan) in PBS for 10 min and blocked in PBS containing 1% BSA for 1 h at room temperature. The cells were then incubated with primary antibodies diluted in PBS+1% BSA at 4°C overnight. After washing with PBS three times, they were incubated with appropriate secondary antibodies diluted in PBS+1% BSA for 2 h. All primary and secondary antibodies are listed in Supplementary Table S2. Nuclei were stained with 4′,6-diamidiono-2-phenylindole (DAPI) (DOJINDO, Kumamoto, Jaoan) for 30 min. They were washed for three times in PBS up to 30–60 min. Fluorescence and bright-field images were captured using a microscope (Ti-U and C-HGFI, Nikon, Tokyo, Japan).

### 2.7 Rhodamine 123 assay

The rhodamine 123 assay was performed. We incubated the cells with Hanks’ balanced salt solution (HBSS) containing 100 µM rhodamine 123 (both from Sigma‐Aldrich, Tokyo, Japan) for 30 min at 37°C and washed them with HBSS twice. To inhibit the transporter activity of multidrug‐resistance protein 1 (Mdr1), we incubated the cells with 20 µM verapamil (Tokyo Chemical Industry Co., Ltd. Tokyo, Japan) at 37°C for 2 h before adding rhodamine 123. Fluorescence and bright-field images were captured using a microscope (Ti-U and C-HGFI, Nikon, Tokyo, Japan).

### 2.8 Definition of the biliary lumen by cell‐tracking staining

Because of the functionality of the hCLiP‐derived biliary‐duct‐like structures, they were able to metabolize a cell‐tracking dye to ascertain the extent of the biliary lumen. Therefore, we used the sequential cell‐tracking staining method to determine the tubular lumen in the induced biliary duct structures. Briefly, the induced biliary structures were incubated with 100 µM rhodamine 123 dye for 10 min at 37°C. Subsequently, the biliary‐duct‐like structures were cultured in BIM‐2 medium (BIM‐1 supplemented with 2% Matrigel) for 48 h, followed by incubation with 10 μM cell tracker orange (CTO, C34551; Invitrogen, Tokyo, Japan) dye for 10 min at 37°C. After washing twice with HBSS, images were captured using a microscope (Ti-U and C-HGFI, Nikon, Tokyo, Japan).

### 2.9 Cholyl-lysyl-fluorescein (CLF) dye assay

We loaded the cells with 1 μM CLF (Corning Life Sciences, Bedford, United States) for 30 min at 37°C and washed them twice with HBSS. We observed the cells and captured images using a confocal microscope. We replaced the cell medium with BIM medium to keep the cells alive for an extended period.

### 2.10 Statistical analysis

Statistical analyses were performed, and graphs were made using GraphPad Prism 8.0 (GraphPad Software Inc., San Diego, CA, United States). Data were analyzed by Student’s t-test or a one-way ANOVA. Details of the statistical analyses and the associated values are described in the respective figure legends.

## 3 Results

### 3.1 FAC medium converted human cryo-hepatocytes into hepatic progenitor cells

**FIGURE 1:**
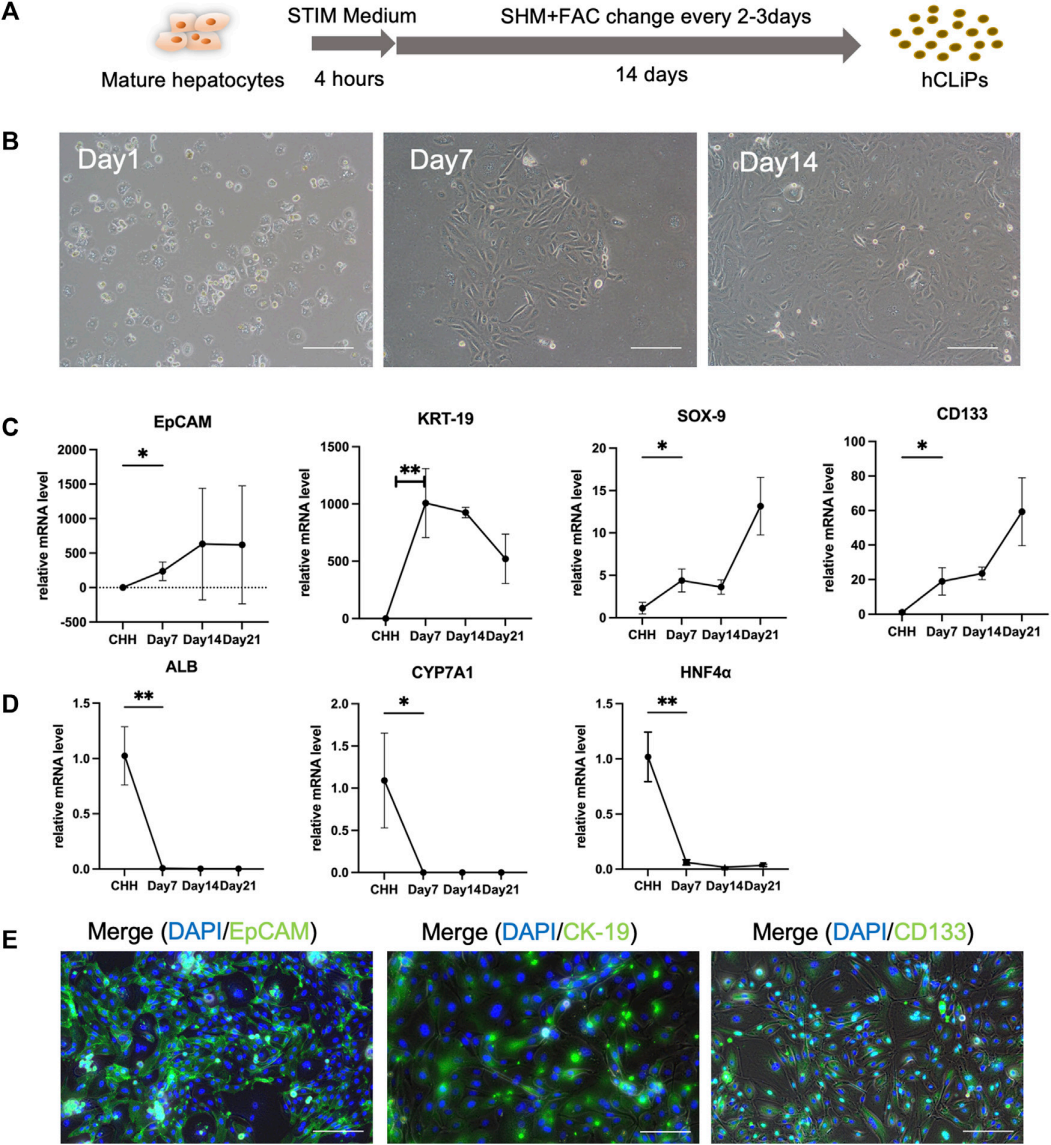
FAC medium converted human cryohepatocytes into hepatic progenitor cells. **(A)** FAC medium could induce human mature hepatocytes (MHs) into chemically induced liver progenitor cells (hCLiPs). **(B)** The morphology of the MHs gradually changed during 2 weeks of FAC culture. Scale bar = 100 μm. **(C, D)** RT-qPCR analysis showed that the hepatic progenitor cell (LPC)-related markers EpCAM, KRT-19, SOX-9, and CD133 gradually increased (*n* = 3–6), while the MH markers ALB, CYP7A1, and HNF-4α gradually decreased, which indicated that MHs were transferred to the hCLiP (*n* = 3–6). Data represent the mean values. Student’s t-test, *p < 0.05, **p < 0.01. **(E)** Immunofluorescence staining of transformed cells was performed on day 14, and the cells expressed the LPC-related markers EpCAM, CK-19, and CD133. Scale bar = 200 μm.

The transformed cells, CHH, were utilized in the experiments and showed significant morphological changes after just 3 days of culture. Notably, the transformed cells displayed a higher capacity for proliferation compared to primary hepatocytes, achieving approximately 90% confluence within 14 days of culture (Figures 1A, B). Additionally, the proliferation of fibroblasts on the heterogeneous culture plate could be observed (Supplementary Figures S1A, B). The mRNAs of the transformed cells were extracted, and RT‒qPCR was used to analyze the gene expression of the hepatocytes and LPCs. The results of Rt-qPCR confirmed that in transformed cells, the expression of LPC-related genes, including EpCAM, KRT-19, SOX-9, and CD133 (Figure 1C), and fibroblast-related genes, including ACTA2, TGF-β2 and MMP2, was gradually upregulated (Supplementary Figure S1C), while the relative gene expression of Alb, CYP7A1 and HNF4α in MHs was gradually downregulated (Figure 1D). Moreover, the transformed cells expressed the LPC protein markers EpCAM, CK-19 and CD133, while the heterogeneous cells expressed the α-SMA protein (Figure 1E; Supplementary Figure S1C). The data suggested that these transformed cells converted from CHH by a small molecule cocktail with FAC medium were LPCs.

### 3.2 HGF promoted BD formation in three-dimensional culture

**FIGURE 2:**
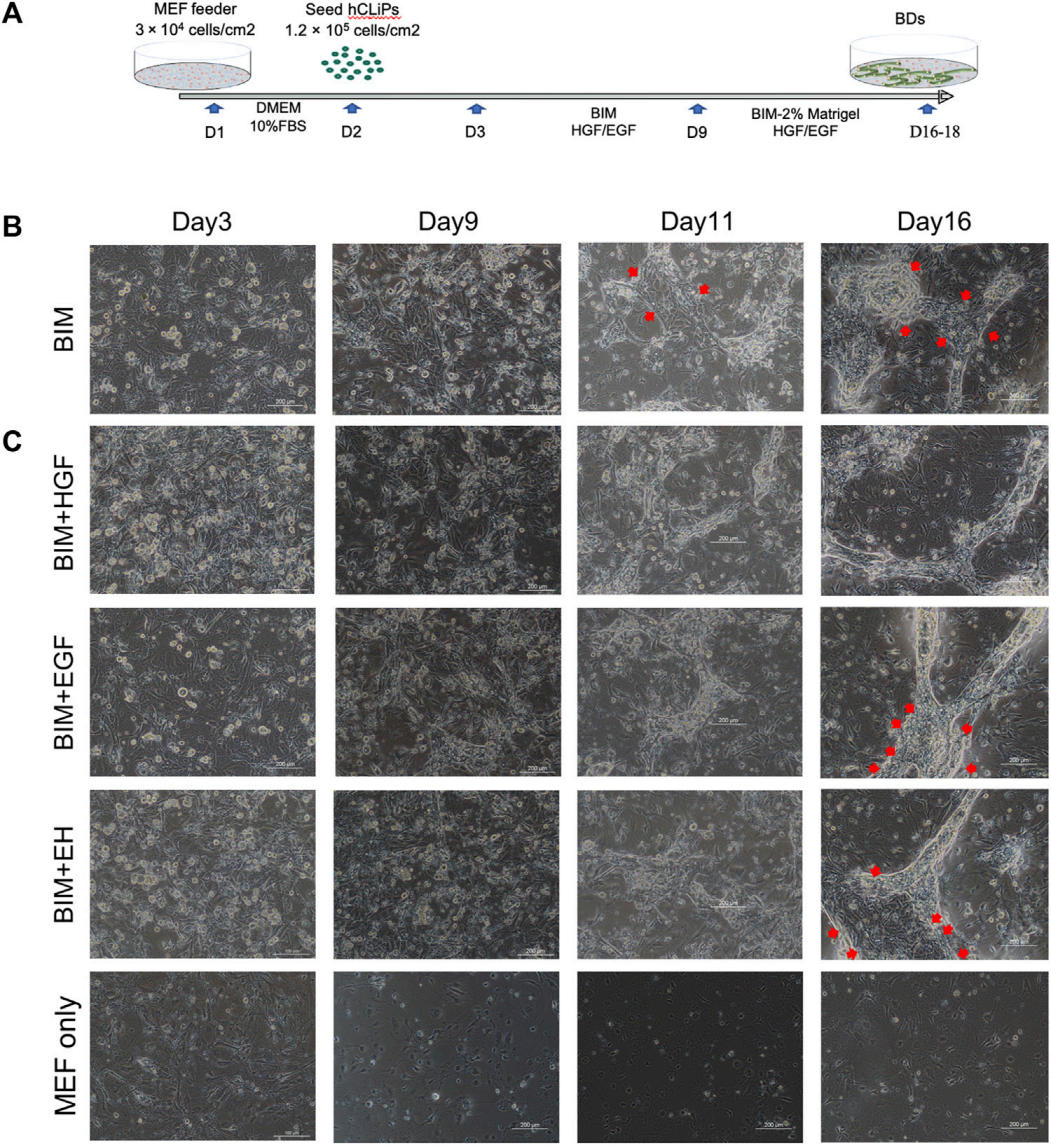
HGF promoted bile duct (BD) formation in three-dimensional culture. **(A)** Embryonic fibroblast feeder cells (MEFs) were used as a layer, and Matrigel was used to construct a three-dimensional culture environment as extracellular matrix (ECM) to induce the hCLiPs to create three-dimensional BDs in a two-step method. **(B)** The bile duct-induced medium (BIM) to induce BD from hCLiP was inefficient (the red arrow shows the untransformed cells). Scale bar = 200 μm. **(C)** In BIM, BIM + EGF, and BIM + EH, BDs were mixed with pieces of hCLiPs or transformed BECs, while BIM with HGF was more effective for forming BDs. Scale bar = 200 μm.

Three-dimensional BD formation was induced by a two-step method in a three-dimensional environment established with MEFs and Matrigel (Figure 2A). Since the direct use of BIM to induce BDs from hCLiPs was not efficient (Figure 2B), we introduced the growth factors HGF and EGF as supplements of BIM. (Tanimizu et al., 2007; Anzai et al., 2016; Kim et al., 2019). In heterogeneous cell culture dishes that induce hepatic progenitor cells, it is imperative to remove a large number of mixed fibrotic cells (Singh et al., 2013). It has been previously reported that many fibrotic cells produced during the induction of MHs are isolated using different culture medium (Miyoshi et al., 2022). When using hCLiPs to generate BDs, fibroblasts affect the formation of BD due to the rapid proliferation of fibroblasts (Supplementary Figure S2A), and the hCLiPs were enriched in purity without damage by multiple subcultures (Supplementary Figure S3A–C). BIM with HGF or EGF could generate BDs in 3D culture earlier (Day 11) in comparison to BIM alone. BIM with HGF was more effective in inducing the transformation of hCLiPs into BECs and the formation of BD tubular structures. In BIM, BIM with EGF, BIM with EH, BD were mixed with pieces of hCLiPs or transformed BECs (Figure 2C).

**FIGURE 3:**
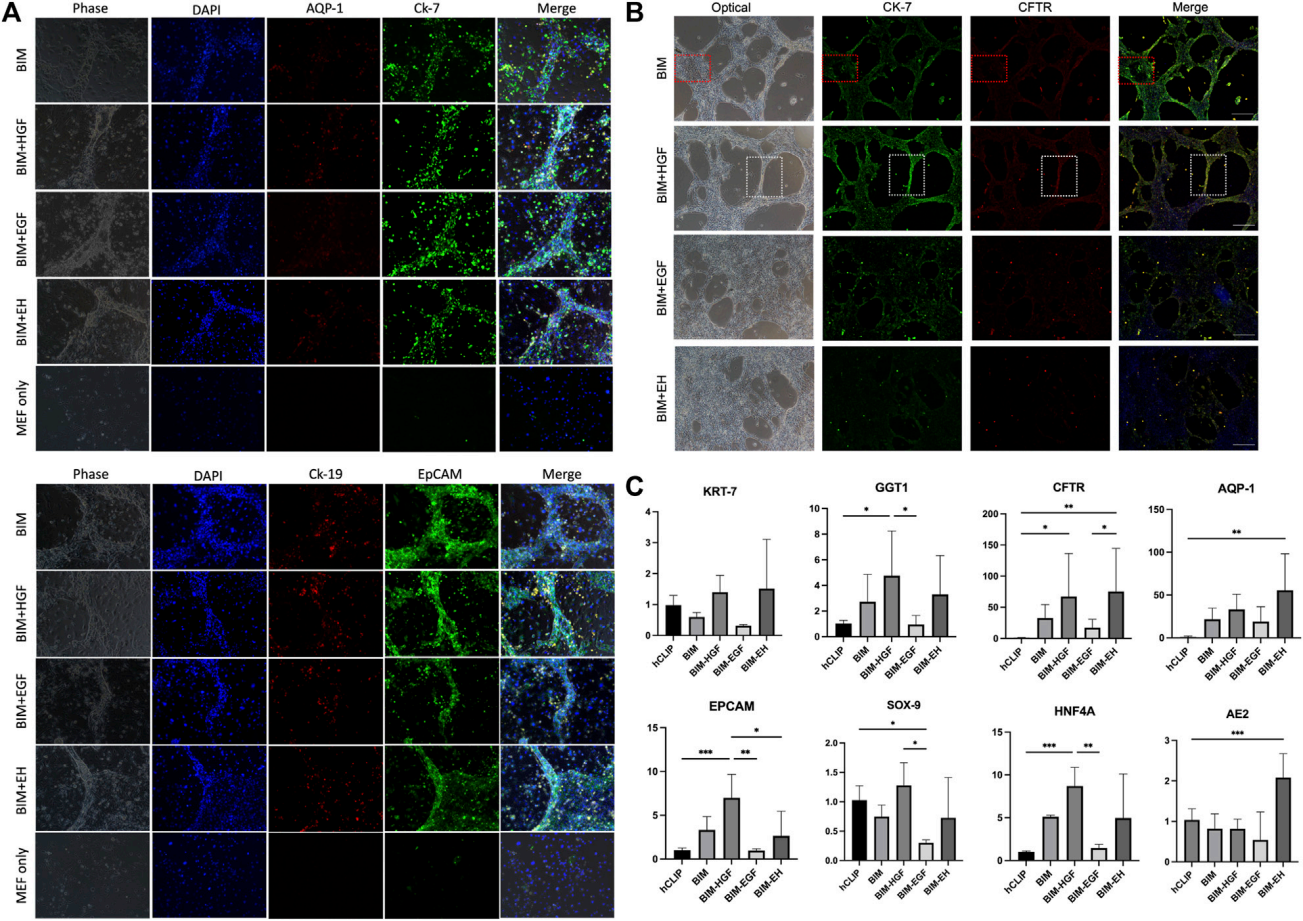
Bile duct-like structures expressed biliary markers. **(A)** The bile duct-related markers AQP-1, CK-7, CK-19, and EpCAM were expressed on all bile ducts. Scale bar = 200 μm.**(B)** The fluorescence intensity of bile duct-related markers CK-7, CFTR, and EpCAM appeared to differ in BIM with HGF, which is more pronounced in areas where bile ducts formed (the red box shows the negative zone, and the white box shows the high-intensity area). Scale bar = 500 μm. **(C)** RT-qPCR was used to assay the bile duct-associated genes KRT-7, GGT1, and CFTR and the progenitor cell-associated genes EPCAM, SOX9, and HNF4A and showed that BIM with HGF increased biliary genes and progenitor genes. (*n* = 3–6) Data represent the mean ± standard error of the mean. One-way ANOVA, *p < 0.05, **p < 0.01.

All four groups were subjected to immunofluorescence microscopy to determine whether the induced biliary-like structure was the BD structure. AQP-1, CK-7, CK-19, and EpCAM (BD-related markers) were expressed on all BDs (Figure 3A), but the fluorescence intensity of CK-7, CFTR, and EpCAM appeared to differ. This was more pronounced in areas where BDs formed (Figure 3B; Supplementary Figures S4). As described above, BD-associated proteins are more pronounced in regions of BD-like formation, and the condition of 3D culture supply with HGF is more helpful in inducing hCLiPs to form 3D BDs. This was also confirmed by RT‒qPCR results for BD-associated genes (KRT-7, GGT1, and CFTR) and progenitor cell-associated genes (EPCAM, SOX9, and HNF4A) (Figure 3C).

### 3.3 The functionality of the BD structure induced by hCLiPs resembled the biliary function

**FIGURE 4:**
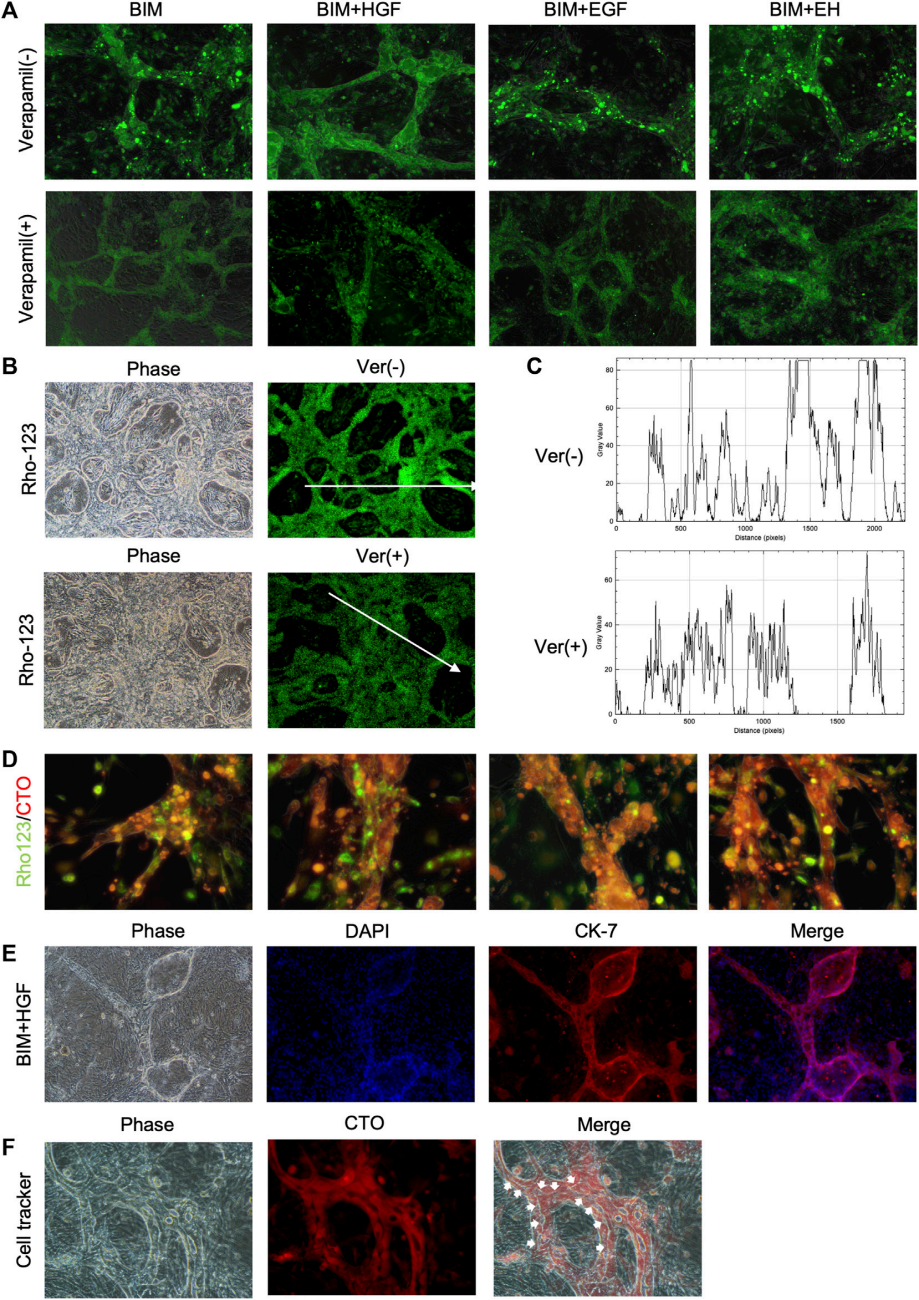
The function of the bile duct structure induced by hCLiPs resembles the biliary function. **(A)** Rhodamine-123 was transported into the BEC and accumulated in the lumen of the biliary duct structures. This could be inhibited by the MDR-1 inhibitor verapamil. **(B, C)** Rhodamine-123 was transported into the BEC and accumulated in the lumen of the biliary duct structures in BIM + HGF group, fluorescence intensity along the line was evaluated by ImageJ. **(D)** To further illustrate the presence of lumen in the bile ducts, after 24 h of staining Rhodamine-123, the dye was taken up by bile duct cells for 30 min using a cell tracker orange. **(E, F)** In the BIM + HGF group, CK-7 showed the structure of the bile duct, and cell-tracker staining analysis (30 min) of the bile duct in the BIM + HGF group showed the lumen in the bile duct.

The functionality of the BD structure was assessed by determining multidrug resistance protein-1 (MDR1) activity, which was evaluated by the ability to transfer rhodamine-123 into the lumen. Rhodamine-123 was transported into the BECs and then accumulated in the lumen of the BD (Figure 4A). In contrast, as the MDR-1 transporter inhibitor, verapamil prevented rhodamine-123 from accumulating in the lumen of the BD, confirming the functional MDR-1 transport activity in the BD (Figure 4A). The bile duct uptake of rhodamine-123 in BIM + HGF group was further examined, and the bile duct uptake of rhodamine-123 could be inhibited by verapamil (Figures 4B, C). To further illustrate the presence of lumen in the BD, after 24 h of staining with rhodamine-123, the dye was taken up by BECs for 30 min using a cell-tracker, and BIM + HGF could be seen to have a more pronounced cavity when viewed under a fluorescence microscope (Figure 4D). In the BIM + HGF group, CK-7 showed the structure of the bile duct with cysts, and 30 min cell-tracker orange staining analysis in the BIM + HGF group showed the lumen in the bile duct in the BIM + HGF group (Figures 4E, F). These results suggested that BIM supplied with HGF could induce the formation of a functional BD with a lumen from hCLiPs.

### 3.4 The human BD structure is integrated with hepatocytes

**FIGURE 5:**
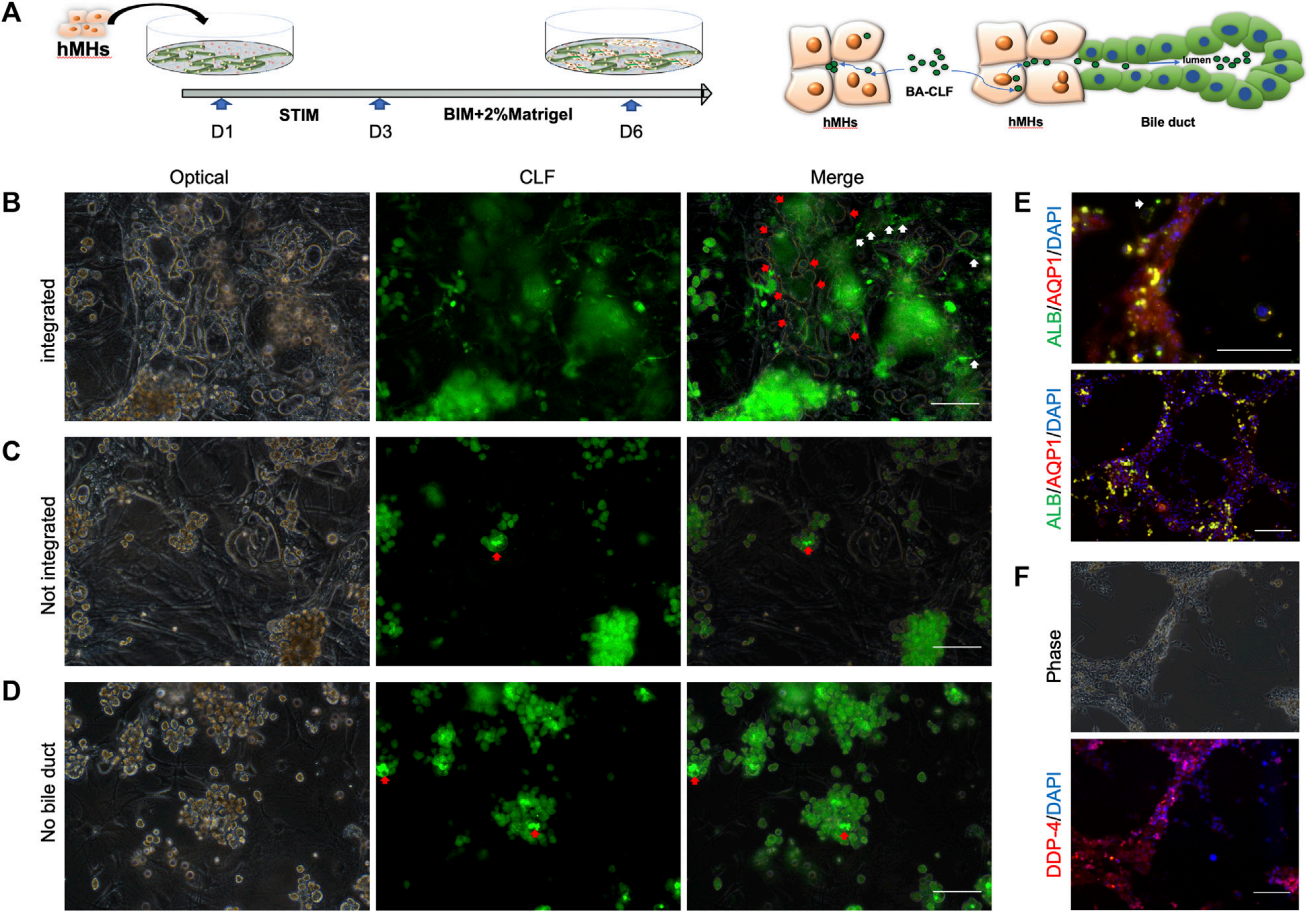
The human bile duct structure is integrated with hepatocytes. **(A)** In the bile duct structures combined with MHs, CLF could accumulate in the lumen from the culture medium through the MHs. Scale bar = 100 μm. **(B, C)** In hepatocytes that were not incorporated into a bile duct or in only MEF conditions, the CLF accumulated in the bile canaliculi formed by the hepatocytes. Scale bar = 100 μm. **(D, E, F)** The immunofluorescence results showed that the integrated MHs expressed albumin (ALB), a marker of mature hepatocytes, dipeptidyl peptidase-4 (DPP-4), a marker protein of the bile canaliculi, and the transporter AQP-1. In contrast, MHs unconnected to the bile duct structures expressed only ALB (the white narrow shows the unintegrated hepatocytes). Scale bar = 200 μm.

The intrahepatic BD forms a complex three-dimensional network configured by cholangiocytes. The BD is responsible for bile acid collection and transplantation from the bile canaliculi among hepatocytes in the *in vivo* hepatic system. To investigate whether the BD structure induced by hCLiPs could collect bile acid from bile canaliculi and hepatocytes, we seeded human hepatocytes into the BD system generated from hCLiPs for 1 day in STIM and 3 days in BIM with HGF, which allowed hepatocytes to integrate into the BD (Figure 5A). Cholyl-l-Lysyl-Fluorescein (CLF) is a fluorescent bile salt derivative that is being developed as an agent for determining the *in vivo* liver function and *in vitro* hepatocyte function (Milkiewicz et al., 2000; de Waart et al., 2010; Huang et al., 2021). BD integrated with hepatocyte tissue could accumulate CLF in the lumen of the BD (Figure 5B). In contrast, the hepatocytes, which did not incorporate BD, accumulated CLF in the bile canaliculi formed by the hepatocytes (Figure 5C). The accumulation of CLF in the bile canaliculi of hepatocytes under MEF-only conditions also confirmed this finding (Figure 5D). Subsequently, immunofluorescence staining of BD-integrated MHs was performed, and the results showed that these MHs expressed albumin (ALB), dipeptidyl peptidase-4 (DPP-4), a marker protein of the bile canaliculi, and the transporter AQP-1 (Figures 5E, F). In contrast, MHs unconnected to the BD expressed only ALB (Figure 5E). The above results showed that the BD integrated the hMH and was able to transport the CLF from the MHs into the BD lumen.

## 4 Discussion

A combination of small molecules could convert MHs into LPCs, which had an unlimited self-renewal capacity and could be induced to differentiate into both MHs and BECs (Katsuda et al., 2017; Katsuda et al., 2019; Kim et al., 2019). In this study, we present a novel *in vitro* three-dimensional BD formed by hCLiPs induced from hMHs, which could combine with hMHs as an integrated tissue with the complete biliary function of the accumulation of the bile analog. This tissue represents a model of the human hepatic organoid that is close to the condition *in vivo*. These results demonstrate the functional properties of BECs and the physiological nature of bile transport in the liver, providing a valuable tool for the study of bile transport and metabolism and related diseases. In addition, this tissue-engineered model holds promise for the development of *in vitro* disease models and drug screening.

Previous studies have explored the use of human BECs *in vitro*; however, their practical application has been limited by a number of factors. The low proportion of BECs in the liver, difficulties in isolation, scarcity of donors, and ethical concerns have all hindered the direct utilization of BECs (Katayanagi et al., 1998; Chen et al., 2018; Huang et al., 2020). The use of small molecules to induce mature cell dedifferentiation has gained increased attention in recent times. Small molecule cocktails do not alter the genetic sequence of the cell, which is different from iPSCs, but instead manipulates the cell fate through alterations in the cell’s epigenetics, offering a straightforward and highly controllable approach (Knyazer et al., 2021; Guan et al., 2022; Hou et al., 2022; Pan et al., 2022). For instance, the three-dimensional BD generated from hCLiPs demonstrated *in vitro* properties that resembled those of intrahepatic BDs, including the transport of the bile analogs CLF and rhodamine-123. Ramli et al. demonstrated the generation of a human hepatic organoid from iPSCs, in which the cells underwent progressive differentiation into hepatocytes and cholangiocytes within approximately 50 days of culture (Ramli et al., 2020).

When isolating mature hepatocytes by two-step perfusion using collagenase, fibrous cells, BD cells and other nonparenchymal cells are always mixed because of the heterogeneity of liver cells (Zhang et al., 2016; Aizarani et al., 2019). Induction cultures are often heterogeneous because of the presence of undifferentiated derivatives and nonparenchymal cells, thereby introducing variability, potential immunogenicity, and problems in directed differentiation (Singh et al., 2013; Miyoshi et al., 2022). When FAC medium induced hepatocyte dedifferentiation, fibroblast cells with strong growth ability grew on P0, gained a growth advantage during the passage process and interfered with the proliferation of hCLiPs after passaging, as shown in the supplemental data. When BDs were induced with hCLiPs without the removal of fibroblasts, the growth of BDs was inhibited by the massive proliferation of fibroblasts during induction. The fibroblast cells from FAC culture caused a high background and limited the growth space. In heterogeneous cell culture dishes that induce hepatic progenitor cells, it is particularly important to remove a large number of mixed fibrotic cells (Singh et al., 2013). The properties of stem cells themselves, which differ in their adhesion to different extracellular matrices, are generally weaker than those of fibrotic cells that are capable of secreting collagen (Wang et al., 2016; Aizarani et al., 2019). Stem cells do not inherently attach to the general surface, and there must be some functionalized surface to facilitate adhesion (Lam and Longaker, 2012). It has been previously reported that a large number of fibrotic cells produced during hepatoprostocyte induction are isolated using different culture media (Miyoshi et al., 2022). It seems feasible to select an appropriate adhesion surface for the separation of different cells, especially stem cells and fibrotic cells with large differences in adhesion ability (Wang et al., 2016). The collagen-coated dish can provide a proper adhesion surface for hCLiPs, but for the gelatin-coated dish, its adhesion ability is significantly weaker than that of ordinary fibroblasts, at least in the adhesion time (Miyoshi et al., 2022). After the one-time fibroblast cell removal procedure, the cells in the final collagen-coated dish were mainly EpCAM-positive cells, with only a small number of Desmin-positive cells. The cells in the gelatin dish were almost all Desmin-positive cells, with few EpCAM-positive cells.

When we attempted to use BIM, which was successfully used for rat BD formation in other studies, to induce directed differentiation of hCLiPs into BECs and subsequently construct three-dimensional BD with medium supplemented with Matrigel, the efficiency of BIM was found to be low (Figure 3) (Huang et al., 2021). The growth factors HGF and EGF were introduced into BIM to improve this situation. Other studies have confirmed that these growth factors promote the induction of three-dimensional BD, especially HGF, which can induce the tubular formation of BDs (Tanimizu et al., 2007; Anzai et al., 2016; Tian et al., 2016; Kim et al., 2019). Dong et al. summarized that the components of liver organoids, including R-spondin-1, forskolin, Wnt, EGF, fibroblast growth factor (FGF), HGF, and TGF-β inhibitors, promote the differentiation of LPCs into BECs. Tanimizu et al. demonstrated that PI3K activated by EGF in combination with HGF promoted proliferation during cyst morphogenesis, and tubular or cyst formation depended on the percentage of Matrigel in the total gel volume. According to some studies, hepatocyte growth factor (HGF) can promote the tube formation of endothelial cells and ductal structure formation of BD cells in three-dimensional culture conditions (Saiki et al., 2006; Tian et al., 2016). HGF can also stimulate the proliferation and differentiation of hepatocytes and induce the formation of hepatocyte-derived biliary epithelium *in vitro* (Limaye et al., 2008; Rose et al., 2021; Tanimizu et al., 2021). The biological responses of scatter, growth, and branching morphogenesis mediated by the HGF receptor are triggered by tyrosine phosphorylation of a single multifunctional docking site located in the carboxy-terminal tail of the receptor (Ponzetto et al., 1994; Boccaccio et al., 1998). The study demonstrated that the HGF receptor binds and phosphorylates Stat-3 and that the ensuing nuclear signaling is required to trigger differentiation for branching morphogenesis (Boccaccio et al., 1998). While HGF, EGF, and the combination of the two induce the transdifferentiation of hepatocytes to BECs, hepatocyte-to-BEC transdifferentiation is regulated by HGF and EGF receptors, and PI3 kinase–mediated signaling independent of AKT is a crucial component of the transdifferentiation process (Limaye et al., 2008). The activation of HGF-Met signaling induces diverse morphogenetic responses, including the formation of branching tubules, cell scattering, and invasion (Birchmeier et al., 2003; Christensen et al., 2005). Therefore, HGF may have a positive effect on BD formation *in vitro* and in three-dimensional culture. Moreover, the LPCs developed cysts with the central lumen in 40% Matrigel, and a lower percentage of Matrigel would form tubular structures (Tanimizu et al., 2007). Tian et al. reported that they had efficiently and controllably generated two-dimensional and three-dimensional BD from iPSC-derived spheroids. The three-dimensional BD structures were formed under control by HGF and EGF in a three-dimensional ECM constructed by Matrigel (Tian et al., 2016). Based on these findings, we compared the efficiency of HGF, EGF, and the combination of the two, and they could promote formation during tubular morphogenesis, but HGF only seemed more efficient. In another similar hCLiPs, Kim et al. reported that hCLiPs formed a tube-like branching morphology using BEC differentiation medium consisting of DMEM/F12 medium containing 10% FBS and 20 ng/mL HGF in a three-dimensional culture constructed with collagen gel (Kim et al., 2019). Indeed, the growth factors used in different culture systems and stem cells are different. Nevertheless, it is undeniable that EGF and HGF are culture components that can be considered to form tubular BD.

Bile is secreted from hepatocytes, extracted into bile canaliculi formed by hepatocytes and subsequently delivered to the intrahepatic BDs, where it is modified by BECs. The BECs form the intrahepatic BD and extrahepatic BD, which complete the modification, secretion, transportation, accumulation, and discharge of bile (Reshetnyak, 2013). Huang et al. and Katsuda et al. demonstrated that they generated rat BD or integrated tubule‐hepatocyte tissues *in vitro* that could transport CLF, rhodamine 123 or fluorescein diacetate (Katsuda et al., 2017; Huang et al., 2021). This is the first reported instance of a tissue connection between the human tubular BD and hepatocytes and presents an excellent opportunity to develop an organoid model for studying diseases related to human biliary hepatocytes. *In vitro*, the functionality of the BD was assessed by determining MDR1 activity, which was evaluated by the ability to transfer rhodamine-123 into the lumen. The BD could collect rhodamine-123 in the BECs, which could accumulate in the lumen, and this could be inhibited by verapamil. Additionally, the P-gp transport function was evaluated by the active transport of rhodamine 123, and verapamil was also an inhibitor of the P-gp function (Wang et al., 2022). Milkiewicz et al. demonstrated that the transportation of CLF is mediated by ATP binding cassette subfamily C members 2 and 3 (ABCC2, 3), which encode canalicular multispecific organic anion transporters 2 and 3 (MRP2/3) (Milkiewicz et al., 2000). The bile canaliculi formed by the MHs without connecting BDs were also observed collecting CLF, while it was reported that this phenomenon was not observed in either the BEC monolayer or the BD without hepatocytes (Huang et al., 2021). Therefore, we speculated that the bile canalicular formed by the MHs formed a connection with BD and had the ability to transport the bile analog CLF. Subsequent immunofluorescence experiments also confirmed the presence of AQP-1 and DPP-4 proteins between the MHs and the BD, which are also bile transportation-related proteins. In conclusion, the connection of the MHs to the BD provided a microstructural basis for the excretion of bile.

Overall, this study provides a promising avenue for establishing a novel *in vitro* system that combines hMHs and BD using HGF and Matrigel. The functional BD, generated from the hCLiPs, displayed characteristics of BDs and were able to accumulate a bile analog, demonstrating their biliary function. By coculturing and connecting hMHs and functional BD, researchers can study various physiological and pathophysiological conditions and evaluate drug responses. The future study of the establishment of disease models from this model could provide a valuable tool for drug development and understanding disease mechanisms.

## Data Availability

The original contributions presented in the study are included in the article/Supplementary Material, further inquiries can be directed to the corresponding author.

## Supplementary material

SUPPLEMENTARY FIGURE S1 It was necessary to remove the fibroblast cells mixed with the hCLiPs. **(A)** The fibroblast cells would increase during the induction of hCLiPs by FAC medium. Scale bar = 500 μm. **(B)** If the fibroblast cells mixed in the culture dish were not removed, the fibroblast cells with rapid proliferation ability would overgrow in the culture dish after passaging. **(C)** Fibroblast-related genes, including ACTA2, TGF- β 2, and MMP2, were gradually upregulated during induction. (*n* = 3–6) Data represent the mean ± standard error of the mean. One-way ANOVA, **p* < 0.01. **(D)** Heterogeneous cells expressed the EpCAM and α-SMA proteins. Scale bar = 200 μm.

SUPPLEMENTARY FIGURE S2 The fibroblast cells proliferated rapidly in a 3D culture environment, making it difficult to form or fragment 3D bile ducts. Scale bar = 500 μm.

SUPPLEMENTARY FIGURE S3 The hCLiPs were enriched in purity without damage by multiple subcultures. **(A)** Multiple subcultures were utilized to remove mixed fibroblast cells in the suspension of hCLiP. **(B)** Immunofluorescence staining of collagen dish-2 and a gelatin dish on day 8. Conversely to the gelatin dishes, fewer Desmin-positive cells were observed in Collagen dish-2 and most cells were EpCAM-positive. **(C)** RT-qPCR results showed that the fibrosis related genes ACTA2, TGF-β2, and MMP2 were more highly expressed in the fiber cells of the gelatin dishes than in the hCLiPs of collagen dish-2, while the LPC marker genes EpCAM, SOX-9, and CD133 were more highly expressed in the hCLiPs. Data represent the mean ± standard error of the mean. Student’s t test, **p* <0.05,**p* < 0.01.

SUPPLEMENTARY FIGURE S4 The immunofluorescence results showed that the EpCAM protein was expressed in the area of bile duct formation, while the EpCAM protein expression was lower in cells that failed to form tubular structures in the BIM+EGF and BIM+EH groups.

## References

[B1] Aizarani N. Saviano A. Sagar, Mailly L. Durand S. Herman J. S. (2019). A human liver cell atlas reveals heterogeneity and epithelial progenitors. Nature 572, 199–204. 10.1038/s41586-019-1373-2 31292543 PMC6687507

[B3] Alpini G. McGill J. M. LaRusso N. F. (2002). The pathobiology of biliary epithelia. Hepatology 35, 1256–1268. 10.1053/jhep.2002.33541 11981776

[B4] Anzai K. Chikada H. Tsuruya K. Ida K. Kagawa T. Inagaki Y. (2016). Foetal hepatic progenitor cells assume a cholangiocytic cell phenotype during two-dimensional pre-culture. Sci. Rep. 6, 28283. 10.1038/srep28283 27335264 PMC4917868

[B5] Birchmeier C. Birchmeier W. Gherardi E. Vande Woude G. F. (2003). Met, metastasis, motility and more. Nat. Rev. Mol. Cell. Biol. 4, 915–925. 10.1038/nrm1261 14685170

[B6] Boccaccio C. Andò M. Tamagnone L. Bardelli A. Michieli P. Battistini C. (1998). Induction of epithelial tubules by growth factor HGF depends on the STAT pathway. Nature 391, 285–288. 10.1038/34657 9440692

[B7] Buisson E. M. Jeong J. Kim H. J. Choi D. (2019). Regenerative medicine of the bile duct: beyond the myth. Int. J. Stem Cells 12, 183–194. 10.15283/ijsc18055 31022996 PMC6657949

[B8] Cao W. Chen K. Bolkestein M. Yin Y. Verstegen M. M. Bijvelds M. J. (2017). Dynamics of proliferative and quiescent stem cells in liver homeostasis and injury. Gastroenterology 153, 1133–1147. 10.1053/j.gastro.2017.07.006 28716722

[B9] Carberry C. K. Ferguson S. S. Beltran A. S. Fry R. C. Rager J. E. (2022). Using liver models generated from human-induced pluripotent stem cells (iPSCs) for evaluating chemical-induced modifications and disease across liver developmental stages. Toxicol Vitro 83, 105412. 10.1016/j.tiv.2022.105412 PMC9296547 35688329

[B10] Chen C. Jochems P. G. M. Salz L. Schneeberger K. Penning L. C. van de Graaf S. F. J. (2018). Bioengineered bile ducts recapitulate key cholangiocyte functions. Biofabrication 10, 034103. 10.1088/1758-5090/aac8fd 29848792

[B11] Christensen J. G. Burrows J. Salgia R. (2005). c-Met as a target for human cancer and characterization of inhibitors for therapeutic intervention. Cancer Lett. 225, 1–26. 10.1016/j.canlet.2004.09.044 15922853

[B12] de Waart D. R. Häusler S. Vlaming M. L. Kunne C. Hänggi E. Gruss H. J. (2010). Hepatic transport mechanisms of cholyl-L-lysyl-fluorescein. J. Pharmacol. Exp. Ther. 334, 78–86. 10.1124/jpet.110.166991 20388726

[B13] Guan J. Wang G. Wang J. Zhang Z. Fu Y. Cheng L. (2022). Chemical reprogramming of human somatic cells to pluripotent stem cells. Nature 605, 325–331. 10.1038/s41586-022-04593-5 35418683

[B14] Hou X. Ma S. Fan W. Li F. Xu M. Yang C. (2022). Chemically defined and small molecules-based generation of sinoatrial node-like cells. Stem Cell. Res. Ther. 13, 158. 10.1186/s13287-022-02834-y 35410454 PMC8996538

[B15] Huang Y. Sakai Y. Hara T. Katsuda T. Ochiya T. Gu W. (2021). Bioengineering of a CLiP-derived tubular biliary-duct-like structure for bile transport *in vitro* . Biotechnol. Bioeng. 118, 2572–2584. 10.1002/bit.27773 33811654

[B16] Huang Y. Sakai Y. Hara T. Katsuda T. Ochiya T. Gu W. L. (2020). Differentiation of chemically induced liver progenitor cells to cholangiocytes: investigation of the optimal conditions. J. Biosci. Bioeng. 130, 545–552. 10.1016/j.jbiosc.2020.07.009 32782195

[B17] Katayanagi K. Kono N. Nakanuma Y. (1998). Isolation, culture and characterization of biliary epithelial cells from different anatomical levels of the intrahepatic and extrahepatic biliary tree from a mouse. Liver 18, 90–98. 10.1111/j.1600-0676.1998.tb00133.x 9588767

[B18] Katsuda T. Kawamata M. Hagiwara K. Takahashi R. u. Yamamoto Y. Camargo F. D. (2017). Conversion of terminally committed hepatocytes to culturable bipotent progenitor cells with regenerative capacity. Cell. Stem Cell. 20, 41–55. 10.1016/j.stem.2016.10.007 27840021

[B19] Katsuda T. Matsuzaki J. Yamaguchi T. Yamada Y. Prieto-Vila M. Hosaka K. (2019). Generation of human hepatic progenitor cells with regenerative and metabolic capacities from primary hepatocytes. Elife 8, e47313. 10.7554/eLife.47313 31393263 PMC6731094

[B20] Kim H. Im I. Jeon J. S. Kang E. H. Lee H. A. Jo S. (2022). Development of human pluripotent stem cell-derived hepatic organoids as an alternative model for drug safety assessment. Biomaterials 286, 121575. 10.1016/j.biomaterials.2022.121575 35598335

[B21] Kim Y. Kang K. Lee S. B. Seo D. Yoon S. Kim S. J. (2019). Small molecule-mediated reprogramming of human hepatocytes into bipotent progenitor cells. J. Hepatol. 70, 97–107. 10.1016/j.jhep.2018.09.007 30240598

[B22] Knyazer A. Bunu G. Toren D. Mracica T. B. Segev Y. Wolfson M. (2021). Small molecules for cell reprogramming: a systems biology analysis. Aging (Albany NY) 13, 25739–25762. 10.18632/aging.203791 34919532 PMC8751603

[B23] Lam M. T. Longaker M. T. (2012). Comparison of several attachment methods for human iPS, embryonic and adipose-derived stem cells for tissue engineering. J. Tissue Eng. Regen. Med. 6 (Suppl. 3), s80–s86. 10.1002/term.1499 22610948 PMC4086291

[B24] Lazaridis K. N. LaRusso N. F. (2015). The cholangiopathies. Mayo Clin. Proc. 90, 791–800. 10.1016/j.mayocp.2015.03.017 25957621 PMC4533104

[B25] Li M. Gong J. Gao L. Zou T. Kang J. Xu H. (2022). Advanced human developmental toxicity and teratogenicity assessment using human organoid models. Ecotoxicol. Environ. Saf. 235, 113429. 10.1016/j.ecoenv.2022.113429 35325609

[B26] Limaye P. B. Bowen W. C. Orr A. V. Luo J. Tseng G. C. Michalopoulos G. K. (2008). Mechanisms of hepatocyte growth factor–mediated and epidermal growth factor–mediated signaling in transdifferentiation of rat hepatocytes to biliary epithelium. Hepatology 47, 1702–1713. 10.1002/hep.22221 18398918 PMC2615562

[B27] Milkiewicz P. Saksena S. Cardenas T. Mills C. O. Elias E. (2000). Plasma elimination of cholyl-lysyl-fluorescein (CLF): a pilot study in patients with liver cirrhosis. Liver 20, 330–334. 10.1034/j.1600-0676.2000.020004330.x 10959812

[B28] Miyoshi T. Hidaka M. Miyamoto D. Sakai Y. Murakami S. Huang Y. (2022). Successful induction of human chemically induced liver progenitors with small molecules from damaged liver. J. Gastroenterol. 57, 441–452. 10.1007/s00535-022-01869-5 35294680

[B29] O'Hara S. P. Tabibian J. H. Splinter P. L. LaRusso N. F. (2013). The dynamic biliary epithelia: molecules, pathways, and disease. J. Hepatol. 58, 575–582. 10.1016/j.jhep.2012.10.011 23085249 PMC3831345

[B30] Olgasi C. Cucci A. Follenzi A. (2020). iPSC-derived liver organoids: a journey from drug screening, to disease modeling, arriving to regenerative medicine. Int. J. Mol. Sci. 21, 6215. 10.3390/ijms21176215 32867371 PMC7503935

[B31] Pan T. Wang N. Zhang J. Yang F. Chen Y. Zhuang Y. (2022). Efficiently generate functional hepatic cells from human pluripotent stem cells by complete small-molecule strategy. Stem Cell. Res. Ther. 13, 159. 10.1186/s13287-022-02831-1 35410439 PMC8996222

[B32] Park Y. Thadasina D. Bolujo I. Isidan A. Cross-Najafi A. A. Lopez K. (2022). Three-dimensional organoids as a model to study nonalcoholic fatty liver disease. Semin. Liver Dis. 42, 423–433. 10.1055/a-1934-5588 36044928 PMC11567686

[B33] Ponzetto C. Bardelli A. Zhen Z. Maina F. dalla Zonca P. Giordano S. (1994). A multifunctional docking site mediates signaling and transformation by the hepatocyte growth factor/scatter factor receptor family. Cell. 77, 261–271. 10.1016/0092-8674(94)90318-2 7513258

[B34] Ramli M. N. B. Lim Y. S. Koe C. T. Demircioglu D. Tng W. Gonzales K. A. U. (2020). Human pluripotent stem cell-derived organoids as models of liver disease. Gastroenterology 159, 1471–1486.e12. 10.1053/j.gastro.2020.06.010 32553762

[B35] Reshetnyak V. I. (2013). Physiological and molecular biochemical mechanisms of bile formation. World J. Gastroenterol. 19, 7341–7360. 10.3748/wjg.v19.i42.7341 24259965 PMC3831216

[B36] Roos F. J. M. van Tienderen G. S. Wu H. Bordeu I. Vinke D. Albarinos L. M. (2022). Human branching cholangiocyte organoids recapitulate functional bile duct formation. Cell. Stem Cell. 29, 776–794.e13. 10.1016/j.stem.2022.04.011 35523140

[B37] Rose S. Ezan F. Cuvellier M. Bruyère A. Legagneux V. Langouët S. (2021). Generation of proliferating human adult hepatocytes using optimized 3D culture conditions. Sci. Rep. 11, 515. 10.1038/s41598-020-80019-4 33436872 PMC7804446

[B38] Saiki A. Watanabe F. Murano T. Miyashita Y. Shirai K. (2006). Hepatocyte growth factor secreted by cultured adipocytes promotes tube formation of vascular endothelial cells *in vitro* . Int. J. Obes. (Lond) 30, 1676–1684. 10.1038/sj.ijo.0803316 16552403

[B39] Sampaziotis F. Muraro D. Tysoe O. C. Sawiak S. Beach T. E. Godfrey E. M. (2021). Cholangiocyte organoids can repair bile ducts after transplantation in the human liver. Science 371, 839–846. 10.1126/science.aaz6964 33602855 PMC7610478

[B40] Sato K. Zhang W. Safarikia S. Isidan A. Chen A. M. Li P. (2021). Organoids and spheroids as models for studying cholestatic liver injury and cholangiocarcinoma. Hepatology 74, 491–502. 10.1002/hep.31653 33222247 PMC8529583

[B41] Si-Tayeb K. Lemaigre F. P. Duncan S. A. (2010). Organogenesis and development of the liver. Dev. Cell. 18, 175–189. 10.1016/j.devcel.2010.01.011 20159590

[B42] Singh A. Suri S. Lee T. Chilton J. M. Cooke M. T. Chen W. (2013). Adhesion strength-based, label-free isolation of human pluripotent stem cells. Nat. Methods 10, 438–444. 10.1038/nmeth.2437 23563795 PMC3641175

[B43] Strazzabosco M. Fabris L. Spirli C. (2005). Pathophysiology of cholangiopathies. J. Clin. Gastroenterol. 39, S90–S102. 10.1097/01.mcg.0000155549.29643.ad 15758666

[B44] Tanimizu N. Ichinohe N. Sasaki Y. Itoh T. Sudo R. Yamaguchi T. (2021). Generation of functional liver organoids on combining hepatocytes and cholangiocytes with hepatobiliary connections *ex vivo* . Nat. Commun. 12, 3390. 10.1038/s41467-021-23575-1 34099675 PMC8185093

[B45] Tanimizu N. Miyajima A. Mostov K. E. (2007). Liver progenitor cells develop cholangiocyte-type epithelial polarity in three-dimensional culture. Mol. Biol. Cell. 18, 1472–1479. 10.1091/mbc.e06-09-0848 17314404 PMC1838984

[B46] Tanimizu N. Nakamura Y. Ichinohe N. Mizuguchi T. Hirata K. Mitaka T. (2013). Hepatic biliary epithelial cells acquire epithelial integrity but lose plasticity to differentiate into hepatocytes *in vitro* during development. J. Cell. Sci. 126, 5239–5246. 10.1242/jcs.133082 24046446

[B47] Tian L. Deshmukh A. Ye Z. Jang Y. Y. (2016). Efficient and controlled generation of 2D and 3D bile duct tissue from human pluripotent stem cell-derived spheroids. Stem Cell. Rev. Rep. 12, 500–508. 10.1007/s12015-016-9657-5 27138846 PMC6186008

[B48] Velazquez J. J. Ebrahimkhani M. R. (2021). Cholangiocyte organoids as a cell source for biliary repair. Transpl. Int. 34, 999–1001. 10.1111/tri.13902 33977592 PMC8562587

[B49] Wang X. Ni C. Jiang N. Wei J. Liang J. Zhao B. (2020). Generation of liver bipotential organoids with a small-molecule cocktail. J. Mol. Cell. Biol. 12, 618–629. 10.1093/jmcb/mjaa010 32232340 PMC7683013

[B50] Wang Y. Zhang W. Yuan J. Shen J. (2016). Differences in cytocompatibility between collagen, gelatin and keratin. Mater Sci. Eng. C Mater Biol. Appl. 59, 30–34. 10.1016/j.msec.2015.09.093 26652345

[B51] Wang Z. Faria J. van der Laan L. J. W. Penning L. C. Masereeuw R. Spee B. (2022). Human cholangiocytes form a polarized and functional bile duct on hollow fiber membranes. Front. Bioeng. Biotechnol. 10, 868857. 10.3389/fbioe.2022.868857 35813994 PMC9263983

[B52] Xu X. Jiang S. Gu L. Li B. Xu F. Li C. (2022). High-throughput bioengineering of homogenous and functional human-induced pluripotent stem cells-derived liver organoids via micropatterning technique. Front. Bioeng. Biotechnol. 10, 937595. 10.3389/fbioe.2022.937595 36032707 PMC9399390

[B53] Yan J. Tai Y. Zhou H. (2022). Culture of mouse liver ductal organoids. Methods Mol. Biol. 2455, 117–129. 10.1007/978-1-0716-2128-8_11 35212991 PMC9327439

[B54] Zhang C. Y. Yuan W. G. He P. Lei J. H. Wang C. X. (2016). Liver fibrosis and hepatic stellate cells: etiology, pathological hallmarks and therapeutic targets. World J. Gastroenterol. 22, 10512–10522. 10.3748/wjg.v22.i48.10512 28082803 PMC5192262

