# Supplementary figures and images for: Three-dimensional human bile duct formation from chemically induced human liver progenitor cells
Source: Front Bioeng Biotechnol. 2023 Aug 21;11:1249769. doi: 10.3389/fbioe.2023.1249769 (PMC10475568; doi:10.3389/fbioe.2023.1249769)

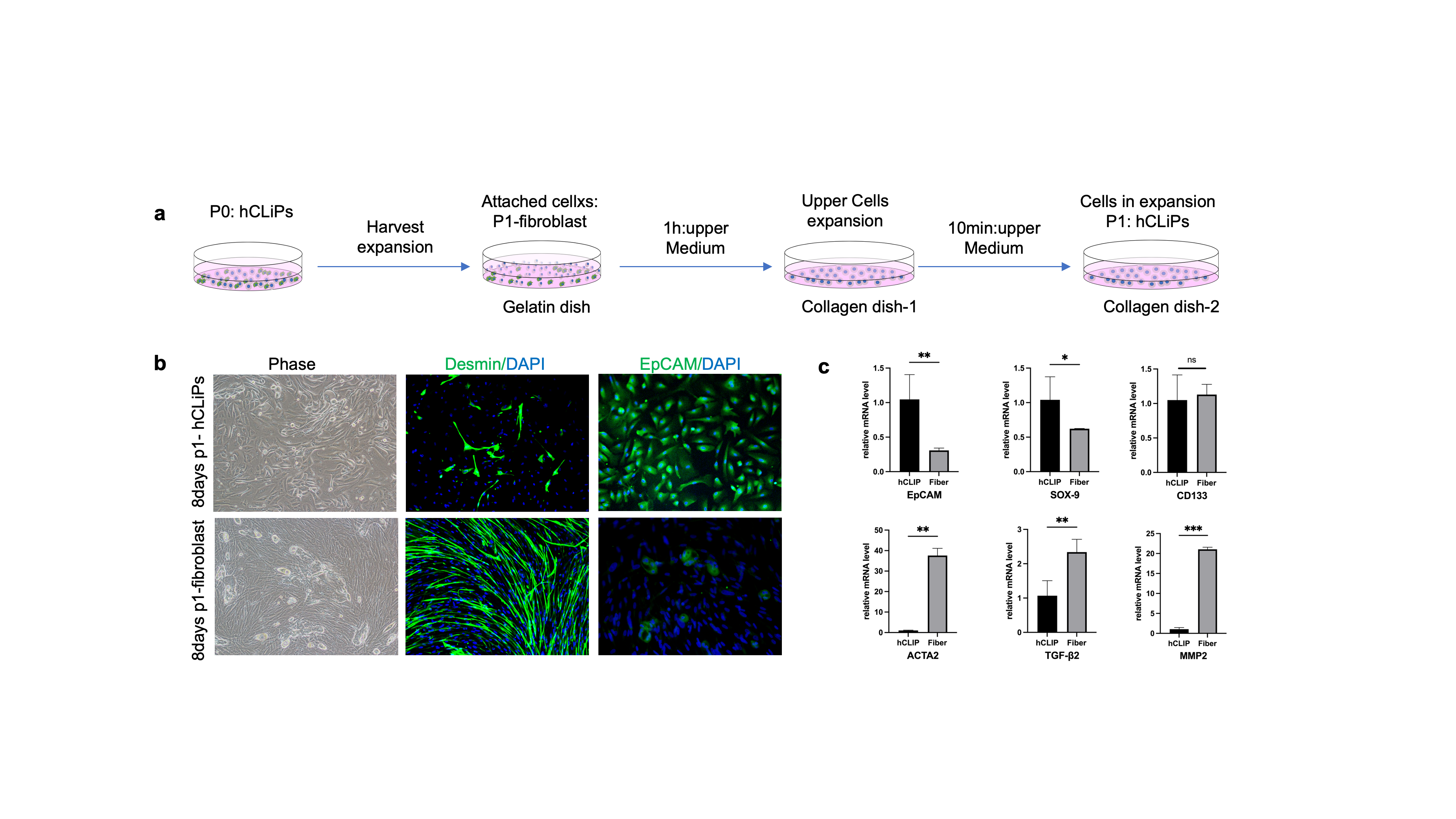

Supplement: Supplementary file 1 [file Image3.tiff]

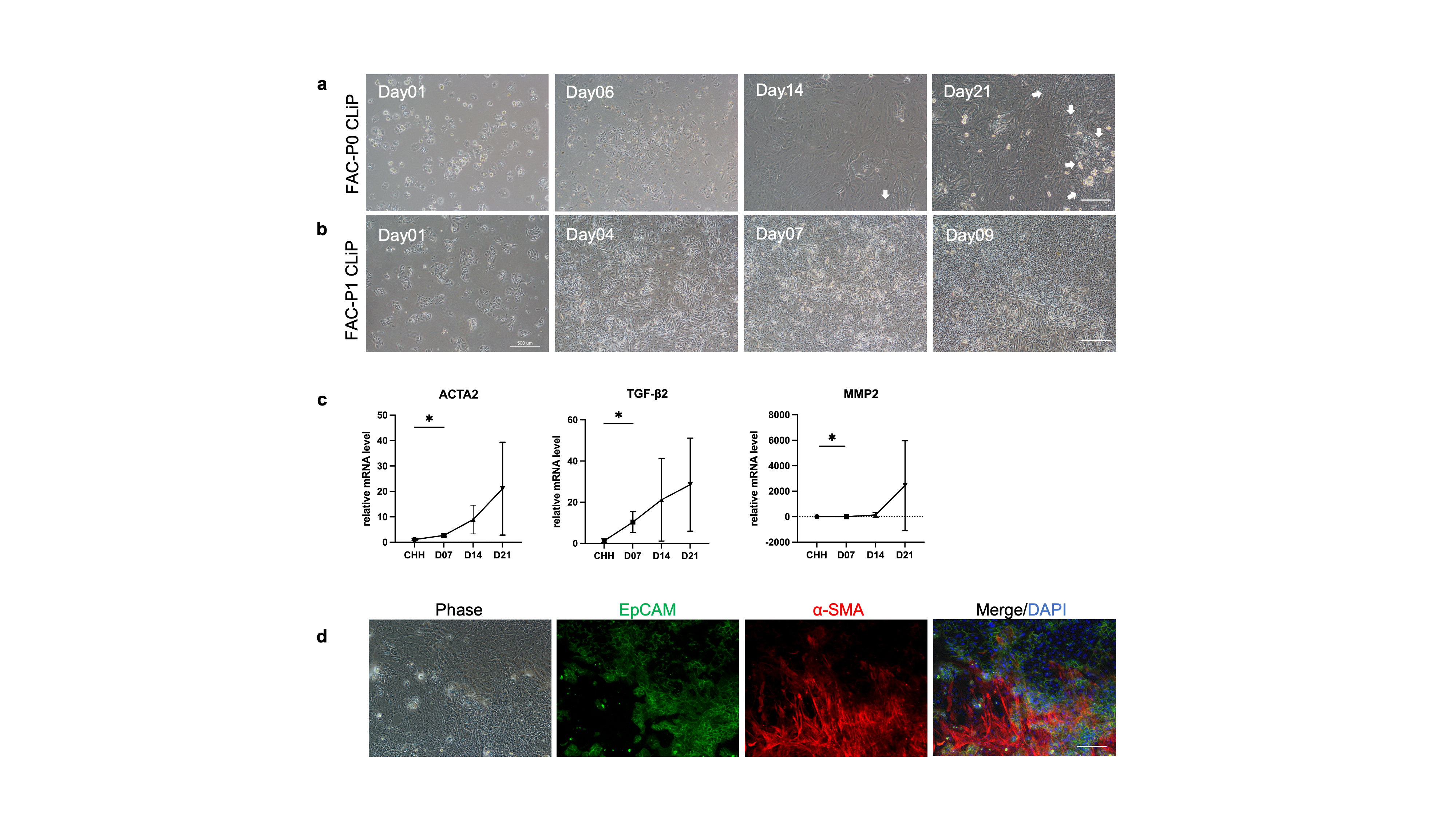

Supplement: Supplementary file 2 [file Image1.tiff]

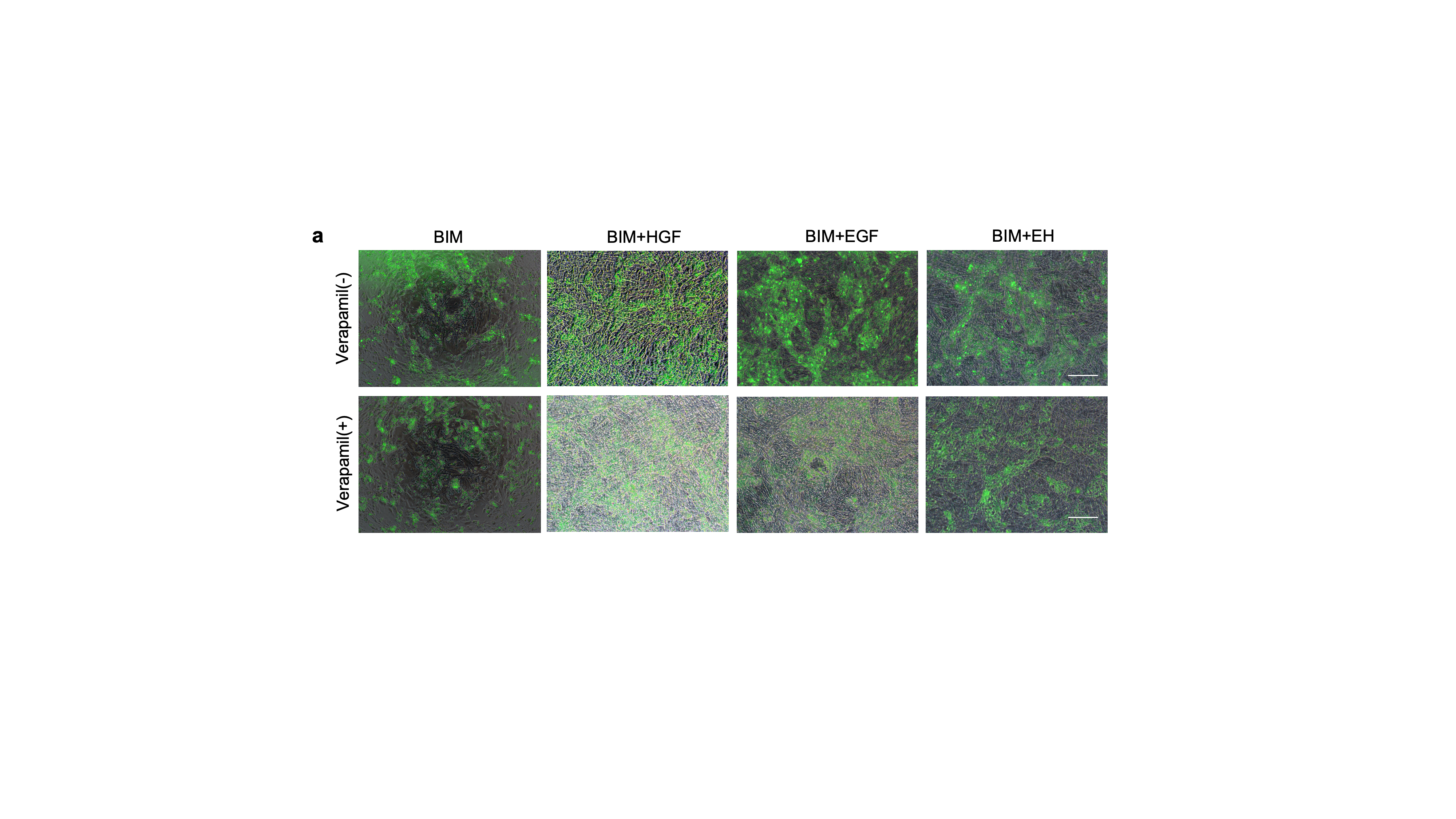

Supplement: Supplementary file 4 [file Image2.tiff]

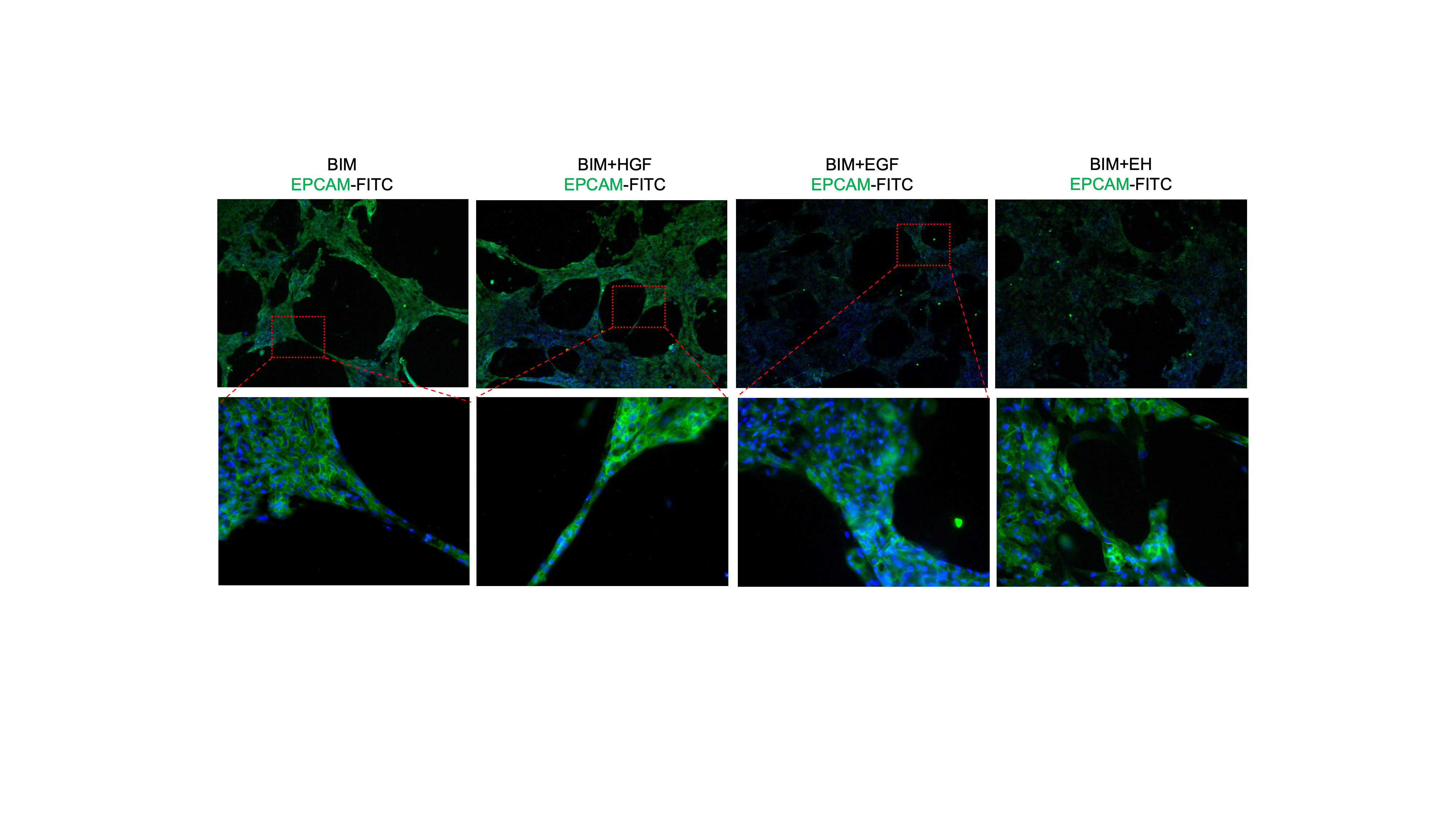

Supplement: Supplementary file 5 [file Image4.tiff]
