# Supplementary material for: Three-dimensional human bile duct formation from chemically induced human liver progenitor cells
Source: Front Bioeng Biotechnol. 2023 Aug 21;11:1249769. doi: 10.3389/fbioe.2023.1249769 (PMC10475568; doi:10.3389/fbioe.2023.1249769)
Supplement: Supplementary file 3 [file Table1.docx]

| Gene Aliases | Gene symbol | Gene name | TaqMan® ID | Amplicon Length | Target species |
| --- | --- | --- | --- | --- | --- |
| GAPDH | GAPDH | Glyceraldehyde-3-Phosphate-Dehydrogenase | Hs99999905_m1 | 122 | Human |
| EpCAM | EpCAM | Epithelial Cell Adhesion Molecule | Hs00901885_m1 | 95 | Human |
| SOX9 | SOX-9 | SRY-Box 9 | Hs00165814_m1 | 102 | Human |
| CD133 | PROM1 | Prominin 1 | Hs01009261_m1 | 106 | Human |
| HNF4A | HNF4A | Hepatocyte Nuclear Factor 4 Alpha | Hs01023298_m1 | 66 | Human |
| AQP-1 | AQP-1 | Aquaporin 1 | Hs01028916_m1 | 96 | Human |
| AE2 | SLC4A2 | Solute Carrier Family 4-Member 2 | Hs01577927_g1 | 103 | Human |
| KRT-19 | KRT-19 | Keratin 19 | Hs00761767_s1 | 116 | Human |
| KRT-7 | KRT-7 | Keratin 7 | Hs00559840_m1 | 95 | Human |
| ALB | ALB | Albumin | Hs00910225_m1 | 137 | Human |
| CFTR | CFTR | Cystic Fibrosis Transmembrane Conductance Regulator | Hs00357011_m1 | 93 | Human |
| CYP7A1 | CYP7A1 | Cytochrome P450 7A1 | Hs00167982_m1 | 66 | Human |
| GGT1 | GGT1 | Gammma-Glutamyltransferase1 | Hs06629878_g1 | 90 | Human |
| α-SMA | ACTA2 | Actin Alpha 2 | Hs00426835_g1 | 105 | Human |
| TGF-β2 | TGF-β2 | Transforming Growth Factor Beta 2 | Hs00234244_m1 | 92 | Human |
| MMP2 | MMP2 | Matrix Metallopeptidase 2 | Hs01548727_m1 | 65 | Human |

**Supplementary Table 1: list of the Taq-man primer of Rt-PCR performed in the experiments.**

**Supplementary Table 2. List of the first and secondary antibodies in the experiments.**

| EpCAM antibody | Ab223582 | Abcam |
| --- | --- | --- |
| CK-7 antibody | Ab181598 | Abcam |
| Desmin antibody | Ab15200 | Abcam |
| AQP-1 antibody | Ab9566 | Abcam |
| DPP-4 (CD26) antibody | Ab28340 | Abcam |
| CK-19 antibody | Ab7755 | Abcam |
| DAPI | Ab15102 | Abcam |
| CD133 | Ab19898 | Abcam |
| CFTR | Ab2784 | Abcam |
| ALB | Ab207327 | Abcam |
| α-SMA | Ab7817 | Abcam |
| Anti-rabbit IgG-peroxidase | A0545 | Sigma-Aldrich Co. LLC. |
| Anti-goat IgG-peroxidase | A5420 | Sigma-Aldrich Co. LLC. |
| Anti-mouse IgG-peroxidase | A9044 | Sigma-Aldrich Co. LLC. |
